# Stronger Is Better: The Impact of Upper Body Strength in Double Poling Performance

**DOI:** 10.3389/fphys.2019.01091

**Published:** 2019-08-23

**Authors:** Arnstein Sunde, Jan-Michael Johansen, Martin Gjøra, Gøran Paulsen, Morten Bråten, Jan Helgerud, Øyvind Støren

**Affiliations:** ^1^Department of Sport and Outdoor Life Studies, University of South-Eastern Norway, Bø, Norway; ^2^Department of Technical and Motor Development, The Norwegian Olympic Sports Center, Oslo, Norway; ^3^Department of Circulation and Medical Imaging, Faculty of Medicine Trondheim, Norwegian University of Science and Technology, Trondheim, Norway; ^4^Myworkout, Medical Rehabilitation Centre, Trondheim, Norway

**Keywords:** cross-country skiing performance, double poling time trial, upper body strength characteristics, maximal strength, peak force, poling contact time, time performance in cross-country skiing, poling frequency

## Abstract

The purpose of the present study was to compare time results from a roller-skiing double poling (DP) time trial with different physiological variables, muscular strength variables, and DP characteristics in both male and female young competitive skiers with the same relative training background. In order to do this, 28 (16 women and 12 men) well-trained 16–25-year-old cross-country skiers from three Norwegian high schools for skiers, as well as local high performance competitive skiers from the South-East of Norway were recruited to participate in the study. All participants were tested for; maximal oxygen uptake in running, Peak oxygen uptake in DP, lactate threshold in DP, DP economy, time to voluntary exhaustion in DP, force analyses in DP, one repetition maximum and power output in pulldown, and leg press and a time trial during DP roller skiing. The results expressed strong correlations between roller skiing time trial performance and maximal strength in pull-down, both independent (*r*_xy_ = −0.83, *p* < 0.01) and dependent (*r*_xy–z_ = −0.50, *p* < 0.02) of sex. Higher maximal upper body strength was related to higher DP peak forces (PF) (*r*_xy_ = 0.78, *p* < 0.02), lower DP frequency (*r*_xy_ = −0.71, *p* < 0.01), and shorter DP contact time (CT) (*r*_xy_ = −0.48, *p* < 0.02). The practical implications of the present study is to acknowledge maximal upper body strength as a performance determining factor in DP. This point at the importance of including maximal strength training in cross-country skiers training programs.

## Introduction

Cross-country skiing is an aerobic endurance sport, and the contribution from the aerobic system is approximately 70 – 95% (Vesterinen et al., 2009; Støren et al., 2014; Hébert-Losier et al., 2017). Aerobic endurance sports demands a high maximal oxygen consumption (VO_2__max_), and a good work economy, which both contributes to a high velocity at lactate threshold (LT) (Costill et al., 1973; Pate and Kriska, 1984; di Prampero, 2003; Sunde et al., 2010; Støren et al., 2014). Cross-country skiing competitions range from intensive sprint with a duration of 2–4 min to distance races of 50 km with a duration of 2–3 h. Five out of six cross-country competitions in the World Cup and the Olympic Games are now mass starts (Skattebo et al., 2016). Mass starts combined with better equipment and track preparation, has led to higher skiing speed in recent years. Higher skiing speed require higher rate of force development and muscular power output (Sandbakk et al., 2014).

Previous studies have focused on determining factors for performance in cross-country skiing and have found strong correlations between VO_2__max_ and performance (Bergh, 1987; Ingjer, 1991; Mahood et al., 2001; Larsson et al., 2002; Alsobrook and Heil, 2009; Ainegren et al., 2013; Sandbakk and Holmberg, 2014). Pellegrini et al. (2018) have shown that high-level skiers have better work economy than regional level skiers. Even though an important factor for work economy is the skiers technical skills, several studies have found improvements in performance corresponding to a better work economy after maximal strength training (Østerås et al., 2002; Mikkola et al., 2007; Losnegard et al., 2011). This is in accordance with other studies performed in other endurance sports, such as cycling (Sunde et al., 2010), running (Paavolainen et al., 1999; Støren et al., 2008; Balsalobre-Fernández et al., 2016), and swimming (Aspenes et al., 2009).

Some previous studies have shown a relationship between maximal strength *per se* and aerobic endurance performance. In Støren et al. (2013) in cycling, no correlation between maximal strength and performance was found. However, some other studies have found correlation between specifically upper body power output and performance in both long distance and sprint cross-country competitions (Rundell, 1995; Rundell and Bacharach, 1995; Gaskill et al., 1999; Nesser et al., 2004; Alsobrook and Heil, 2009; Stöggl et al., 2011; Carlsson et al., 2016). Also, Stoggl et al. (2010) have found lean trunk mass to correlate to maximal DP speed.

Double poling (DP) is a high-speed cross-country skiing technique. Total racetime now contains a much larger percentage of DP than only a few years ago (Holmberg et al., 2005; Losnegard et al., 2013). DP is also used in more uphill terrain than before. Hoffman and Clifford (1992) found that DP was a more economical technique than kick DP in flat terrain. DP is thus considered a strong performance-determining technique in classic cross-country skiing.

In previous studies on DP in cross-country skiers, some DP characteristics that are linked to maximal muscular strength have been identified (Bilodeau et al., 1995; Holmberg et al., 2005; Stöggl et al., 2007, 2011; Stöggl and Holmberg, 2011, 2016; Danielsen et al., 2015). Although Stöggl et al. (2011) found correlations between power output in bench press and bench pull, and maximal speed in DP, it was first and foremost the timing and instant of force application that accounted for the inter-individual differences. Several studies have found that the fastest skiers produced the highest peak pole forces (Bilodeau et al., 1995; Holmberg et al., 2005; Stöggl and Holmberg, 2011, 2016). Also, Bilodeau et al. (1995) found the fastest skiers to reach their PF in the shortest time, and hypothesized that the differences in maximal skiing velocity were due to differences in muscular strength. Zoppirolli et al. (2015) found high-level skiers to have a lower DP frequency at the same load as regional-level skiers. Holmberg et al. (2005) found faster skiers to have a shorter propulsion phase and a longer recovery phase when DP at high velocity. Holmberg et al. (2005) also found that the fastest skiers had higher peak pole forces. In contrast to Hoff et al. (1999) and Holmberg et al. (2005) did not find any positive correlation between time to PF and performance in DP. Both Stöggl et al. (2007, 2011), Stöggl and Holmberg (2011), and Jonsson et al. (2019) found that faster skiers produced longer DP cycle lengths (meters) at equal DP frequency, than slower skiers. It may be hypothesized that this was aligned to greater muscular power output.

Male and female athletes at the same relative performance level show sex differences in both VO_2__max_ (Sandbakk et al., 2014; Stöggl et al., 2019), and maximal muscular strength (Sandbakk et al., 2018). Also greater sex differences have been found in exercises where the upper body is involved (Sandbakk et al., 2014). It is thus crucial to evaluate the relative importance of aerobic endurance variables, muscular strength variables, and DP characteristics on DP performance both independent and dependent of sex. A cohort of competitive cross-country skiers from both sexes with the same relative training background and age, but with heterogeneity in performance level would thus be preferable.

The purpose of the present study was therefore to compare roller-skiing time trial (TT) performance with different physiological variables, muscular strength variables, and DP characteristics in both male and female young competitive skiers with the same relative training background. The hypothesis was that maximal upper body strength would significantly impact DP characteristics and performance.

## Materials and Methods

### Approach to the Problem

The main objective of this cross-sectional study was to evaluate correlations between performance in DP cross-country roller skiing and different physiological variables, muscular strength variables, and DP characteristics in both male and female young competitive skiers with the same training background. Comparisons between male and female skiers as well as correlation analyses both independent of and corrected for sex, were thus performed.

### Subjects

A total of 28 (16 women and 12 men) well-trained 16–25-year-old cross-country skiers from three Norwegian high schools for skiers, as well as local high performance competitive skiers from the South-East of Norway participated in this study (Table 1). The study was approved by the institutional research board at the University of South-Eastern Norway (former University College of South-East Norway), and conducted in accordance with the Helsinki declaration. All skiers gave their written consent to participate, after having received information about the study. Parents and coaches to participants under 18 years, also gave their written consent.

**TABLE 1:** Characteristics of skiers (*N* = 28).

	**All (*N* = 28)**		**Females (*N* = 12)**		**Males (*N* = 16)**	
	**Mean ± SD**	**CV (%)**	**Mean ± SD**	**CV (%)**	**Mean ± SD**	**CV (%)**
BW (kg)	70.1 ± 7.5	10.7	65.9 ± 5.2	7.9	73.2 ± 7.5^∗∗^	10.2
Age (years)	18.5 ± 1.3	7.0	18.3 ± 1.2	6.6	18.6 ± 1.4	7.5
VO_2max_ running						
Ml⋅kg^–1^⋅min^–1^	64.0 ± 10.4	16.3	53.8 ± 4.4	8.2	72.2 ± 5.1^∗∗^	7.1

*Values are mean ± standard deviation (SD) and coefficient of variance (CV). BW, body weight; Kg, kilograms; VO_2max_, maximal oxygen uptake; Ml⋅kg^–1^⋅min^–1^, milliliters per kilogram BW per minute. ^∗∗^*p* < 0.01 different from females.*

### Test Procedures

In order to evaluate physiological and technical variables related to performance in DP, the following tests were carried out; VO_2__max_ running, VO_2__peak_ DP, LT in DP, DP economy (C_DP_), time to voluntary exhaustion in DP in the ramp VO_2__peak_ test, force analyses in DP, one repetition maximum (1RM) and power output in pulldown and leg press, and performance during a DP roller skiing time trial (TT).

The skiers were tested over two consecutive days. Day one consisted of an incremental VO_2__max_ test in running and a DPTT test with 1-h rest in between. The subjects started at an intensity of 8–12 km⋅h^–1^ and a 6% inclination. Every 30 s the inclination increased by 1% until 8% inclination was reached. Then the speed was increased by 0.5 km⋅h^–1^ every 30 s. The test terminated at voluntary fatigue, and additionally heart rate (HR) ≥98% of HR_max_, respiratory exchange ratio (RER) ≥1.05, as well as a plateau of the VO_2_ curve was used to evaluate if VO_2__max_ was obtained (Åstrand et al., 2003). All VO_2_ measurements were made by the metabolic test system, Metalyzer II Cortex (Biophysic GmbH, Leipzig, Germany), with a mixing chamber. The treadmill used for running was a Woodway PPS 55 sport (Waukesha, WI, United States). All HR measurements were made by Polar s610 HR monitors (Kempele, Finland).

The double poling time trial test took place in a paved roller ski course track of 940 m with a height difference of 11 m. The subjects completed six laps, totaling 5640 m. This test was organized as an interval start with 30 s between each subjects. The subjects were told to use the DP technique throughout the whole test, and drafting was not allowed (using cycling TT rules). In this test, differences in temperature and humidity in between test days, may lead to differences in rolling resistance. Therefore, we performed a calibration test to calculate a correction factor. One of the test leaders conducted a 50 m roller-timing test with the same roller skis immediately after the time trial test every test day. The test was conducted in a tucked position, with the same test person every day, in a gentle slope, approximately 10%, and with time measured by use of photocell equipment (Musclelab system, Ergotest Inovation, Porsgrunn, Norway). Ten runs were performed for each test, ensuring proper warm up of the wheels, and the average time of the last three runs was used to calculate the correction factor.

The second day of testing consisted of a DP test on a cross-country skiing treadmill, (Rodby RL 2700E, Rodby Innovation, Vänge, Sweden) and two maximal strength tests with 1-h rest in between. The subjects were acquainted to the cross-country skiing treadmill by use of a 30-min workout ahead of the pretest. The first 15 – 20 min consisted of 3–5 four-minute submaximal work periods. Whole blood lactate concentration was measured with a Lactate Scout+ (SensLab GmbH, Leipzig, ray Inc., Kyoto, Japan). Then C_DP_, force measurements and DP characteristics were evaluated. By use of a force transducer, measurements of force and DP characteristics, were possible. The force transducers were integrated in the poles and is a part of the Musclelab system (Ergotest Innovation, Porsgrunn, Norway). The dimension of the force transducer was 4 cm of length and 2 cm in diameter, placed 8 cm below the grip bar, as an integrated part of the pole. Outside the force transducer, a sender with the dimension 4 cm × 4.6 cm × 1 cm was placed. The total weight of the system added 100 g to the pole. The sender communicated by a Nordic semiconductor Gazell stack with a 2.4 GHz band (Nordic Semiconductor, Norway) with the Musclelab system, with a sampling rate of 200 Hz and a resolution of 14 bits. Over all accuracy was 0.9% of full scale. Test retest reliability was checked at our lab, exhibiting a standard error mean of <1%.

The system was calibrated by use of two different external weight loads on top of the pole placed in a vertical position, while the other end of the pole was placed on the force platform for a secondary control. The reading from the sensor of the pole unloaded was recorded and then the reading from the sensor of the pole with external load was recorded. Force was then computed using the formula F = (signal – offset) gain. The subjects started at a work intensity assumed to represent 50 – 70% of their VO_2__peak_ in DP, corresponding to 4% inclination and 11.5 km⋅h^–1^ for men and 6 or 7 km⋅h^–1^ for women. Every 4 min after the first step, the speed was increased by 1–3 km⋅h^–1^, until the protocol terminated at a lactate level above the subjects’ LT. LT was defined as the warm up lactate value (i.e., the lowest measured lactate value) + 2.3 mmol L^–1^. LT was expressed as the VO_2_ in% of VO_2__peak_ DP (%VO_2__peak_), whereas the velocity at LT was expressed as km.h^–1^. This is in accordance with the protocol proposed by Helgerud et al. (1990), using warm up lactate value + 1.5 mmol L^–1^ with the YSI apparatus. As the constant difference in [La−]_b_ between whole blood and hemolyzed blood is 40%, the 1.5 mmol L^–1^ measured by YSI equals 2.3 mmol L^–1^ measured by Lactate Scout+. The advantage of using individual warm-up values compared with e.g., a fixed 4 mmol L^–1^, is that this is less vulnerable to day-to-day variations in subjects [La−]_b_, as previously discussed in Støren et al. (2014). The force measurements as well as the oxygen consumption measurements for calculating C_DP_, were made between minute 3:00 and 03:20 in each work period. The force transducer measured force through the poles, DP frequency, and CT. C_DP_ was calculated as oxygen consumption at LT. All DP characteristic measurements were performed at the same relative intensity, i.e., at LT velocity.

Maximal aerobic speed (MAS) was calculated based on the oxygen consumptions measured in the submaximal work periods and the VO_2__peak_ in DP, and was defined as the velocity where the horizontal line representing VO_2__peak_ meets the extrapolated linear regression representing the sub maximal VO_2_ measured in the LT assessment. The same method was used for cycling in Sunde et al. (2010) and in running in Helgerud et al. (2010), with *r* > 0.99 for the regression lines. MAS equals thus VO_2__peak_DP/C_DP_. Since $\frac{\mathrm{ml}\cdot {\mathrm{kg}}^{-1}\cdot \min^{-1}}{\mathrm{ml}\cdot {\mathrm{kg}}^{-1}\cdot m^{-1}}=\frac{1\cdot \min^{-1}}{1\cdot m^{-1}}=\frac{\text{m}}{\text{min}}$, VO_2peak_ DP/C_DP_ is expressed as a velocity (m min^–1^).

One minute after the last submaximal work period, the subjects carried out an all-out test where time to exhaustion and VO_2__peak_ in DP were measured. This test was implemented as an incremental ramp protocol. The output speed was set to 11.5 and 6 km⋅h^–1^ for men and women, respectively. The inclination was set to 6%, and remained constant through the whole test. The speed was increased by 1 km⋅h^–1^ every 30 s until 18 km⋅h^–1^ (men) and 10 km⋅h^–1^ (women) were reached. The speed was then increased by 0.5 km⋅h^–1^ every 30 s until voluntary exhaustion. The subjects were encouraged to perform their best. Voluntary exhaustion was defined as the point where the subjects could no longer manage to keep the position at the treadmill, but slowly moved backward reaching a pre-defined mark 1 m behind their original position on the mill. The time to exhaustion was registered and the mean of the two subsequent highest registered VO_2_-values, each representing 10 s intervals by the mixing chamber, was representing VO_2__peak_ DP.

After a rest period of minimum 60 min, the subjects were then tested for 1RM and power output in leg press (OPS161 Interchangeable leg press, Vertex, United States), and pulldown (Gym 2000, Vikersund, Norway). From pilot testing in Støren et al. (2008), and later presented in Sunde et al. (2010), no deterioration in 1RM squat was detected 30 min after VO_2__max_ and MAS testing in running and cycling, compared to 1RM without these prior tests. Leg press was chosen as a measure of lower body maximal strength for several reasons. More specialized DP related exercises such as hip flexion involves both the lower body, truncus and to some extent the upper body. The more specialized the exercises, the more practice is needed to perform valid and reliable 1RM tests. Only two maximal strength tests, one for the upper body and one for the lower body were chosen, due to the large total number of tests in this study.

Each lift was performed with a controlled slow eccentric phase, a complete stop of movement for approximately 1 s in the lowest position (leg press) or the highest position (pulldown), followed by a maximal mobilization of force in the concentric phase. The measurements of lifting time, distance of work, and thus power output were performed using the Muscle Lab system (Ergotest Innovation AS, Porsgrunn, Norway). Sensors were placed vertically below the center of the weight loads in both leg press and pulldown, and also at the actual center of the weight loads. Each strength test started using 10 reps at a weight load assumed to be approximately 50% of 1RM. After 3 min of rest: 5 reps at approximately 60% 1RM, then 3 reps at approximately 70% 1RM, 2 reps at approximately 80% and at least 1 rep at estimated 1RM with 3 min rest in between. From there on: 1 rep at a weight load increased by 2.5 – 10 kg from the subsequent lift, followed by 3 min of resting, until reaching 1RM. The time spent in each lift, as well as the work distance was measured. As the external force of each lift is represented by the weight of the lifted bars, the power output can be calculated and expressed as N m s^–1^ or watt (W).

### Statistical Analyses

Normality was tested by use of -plots and found to represent normal distributions for the main variables (TT performance, maximal strength and VO2_peak_ DP). Values were thus expressed descriptively as mean ± SD. Inter-individual variability was expressed as coefficient of variance (CV). Correlations were expressed as the correlation factor *r* from Pearsons bivariate tests. Based on the correlation coefficient definitions by Hopkins (2000), *r* values of 0.3–0.5 = moderate, 0.5–0.7 = large, 0.7–0.9 = very large, 0.9 = nearly perfect, and 1.0 = perfect. We have therefor defined strong correlations to be *r* > 0.7 in the present study. However, as the cohort includes both male and female skiers, partial correlation analyses were also performed corrected for sex. The correlation factor in normal correlations independent of- or within sex has thus been denoted *r*_xy_, whereas the correlation factor in partial correlations corrected for sex has been denoted *r*_xy–z_. The practical (clinical) implication of the relations displayed by the *r* values, were evaluated by use of standard error of the estimate (SEE). This SEE values were obtained from linear regression analyzes. To investigate differences between males and females, independent sample t-tests were performed. Statistical analyzes were performed using the software program statistical package for social science version 24 (SPSS, IBM, Chicago, IL, United States). A *p* value <0.05 was accepted as statistically significant in all tests.

## Results

Test results in the different variables are presented both as total and per sex in Table 2. TT performance was 23% (*p* < 0.01) better in males than in females, and males where 34% (*p* < 0.01) stronger in pulldown than females.

**TABLE 2:** Test results (*N* = 28).

	**All (*N* = 28)**		**Females (*N* = 12)**		**Males (*N* = 16)**	
	**Mean ± SD**	**CV (%)**	**Mean ± SD**	**CV (%)**	**Mean ± SD**	**CV (%)**
TT_DP_ (s)	899.4 ± 152.7	17.0	1032.8 ± 134.6	13.0	799.4 ± 61.5^∗∗^	7.7
**VO_2max_ running**						
L⋅min^–1^	4.48 ± 1.0	22.3	3.54 ± 0.39	11.0	5.22 ± 0.63^∗∗^	12.1
Ml⋅kg^–1^⋅min^–1^	64.0 ± 10.4	16.3	53.8 ± 4.4	8.2	72.2 ± 5.1^∗∗^	7.1
Ml⋅kg^–0.67^⋅min^–0.67^	259.6 ± 46.4	17.9	213.9 ± 17.9	8.4	296.2 ± 22.8^∗∗^	7.7
**VO_2peak_ DP**						
L⋅min^–1^	3.93 ± 0.89	22.6	3.10 ± 0.36	11.6	4.54 ± 0.62^∗∗^	13.7
Ml⋅kg^–1^⋅min^–1^	55.7 ± 9.1	16.3	47.3 ± 5.2	11.0	62.0 ± 5.3^∗∗^	8.5
Ml⋅kg^–0.67^⋅min^–0.67^	226.6 ± 40.5	18.2	188.0 ± 19.8	10.5	255.5 ± 24.1^∗∗^	9.4
Fract util DP (%VO_2max_)	86.9 ± 7.3	7.9	87.9 ± 5.9	6.7	86.0 ± 8.4	9.8
**C_DP_**						
Ml⋅kg^–1^⋅meter^–1^	0.183 ± 0.023	12.6	0.192 ± 0.020	10.4	0.177 ± 0.023	13.0
Ml⋅kg^–0.67^⋅meter^–1^	0.742 ± 0.087	11.7	0.763 ± 0.084	11.0	0.727 ± 0.089	12.2
MAS (km h^–1^)	18.6 ± 4.1	22.0	14.9 ± 1.7	11.4	21.4 ± 3.0^∗∗^	14.0
**LT**						
%VO_2peak_	79.0 ± 9.0	11.3	81.1 ± 4.3	5.3	78.3 ± 11.4	14.6
Km⋅h^–1^	14.6 ± 2.7	18.5	12.1 ± 1.2	9.9	16.5 ± 1.7^∗∗^	10.3
Km⋅h^–1^ calc. (MAS⋅%VO_2peak_)	14.6 ± 2.7	18.5	12.1 ± 1.3	10.0	16.5 ± 1.8^∗∗^	10.4
**Force_DP_**						
Peak (N)	381.8 ± 124.0	32.5	277.6 ± 80.7	28.8	459.9 ± 87.7^∗∗^	19.1
Average during CT (N)	169.3 ± 47.8	28.2	145.6 ± 37.7	25.9	187.1 ± 47.7^∗∗^	25.5
RFD (N⋅s^–1^)	2620 ± 1233	47.0	2063 ± 1230	59.7	3038 ± 1091^∗^	35.9
**DP**						
Freq. at LT (St⋅meter^–1^)	0.239 ± 0.055	23.0	0.286 ± 0.047	6.5	0.204 ± 0.027^∗∗^	13.2
Freq. at LT (St⋅s^–1^)	0.938 ± 0.100	10.7	0.956 ± 0.137	14.3	0.925 ± 0.060	6.5
CT (s)	0.353 ± 0.073	20.7	0.388 ± 0.092	23.7	0.327 ± 0.040^∗^	12.2
**Maximal strength**						
1RM pull-down (kg)	86.2 ± 19.3	22.4	66.0 ± 8.8	13.2	99.7 ± 10.3^∗∗^	10.3
1RM leg-press (kg)	278.1 ± 54.9	19.7	235.6 ± 37.5	15.9	303.7 ± 47.8^∗∗^	17.0
Power pull-down (W)	439.2 ± 122.3	27.8	323.1 ± 54.1	16.7	516.5 ± 87.9^∗∗^	17.0
Power leg-press (W)	609.7 ± 157.2	25.8	466.6 ± 70.2	15.0	694.0 ± 130.3^∗∗^	18.7

*Values are mean ± standard deviation (SD) and coefficient of variance (CV). TT_*DP*_, double poling time trial on roller skies; S, seconds; BW, body weight; Kg, kilograms; VO_2max_, maximal oxygen uptake; L⋅min^–1^, liters per minute; Ml⋅kg^–1^⋅min^–1^, milliliters per kg BW per minute; Ml⋅kg^–0.67^⋅min^–0.67^, milliliters per kg BW raised to the power of 0.67 per minute; DP, double poling; VO_2peak_, peak oxygen uptake during DP; Fract Util, fractional utilization of VO_2peak_ vs. VO_2max_; C_*DP*_, oxygen cost of DP at LT; Ml⋅kg^–1^⋅meter^–1^, milliliters per kg BW per meter; Ml⋅kg^–0.67^⋅meter^–1^, milliliters per kg BW raised to the power of 0.67 per meter; MAS, maximal aerobic speed calculated as peak oxygen uptake during DP divided on C_*DP*_; km⋅h^–1^, kilometers per hour; LT, lactate threshold; N, Newton; CT, contact time; RFD, rate of force development; N⋅s^–1^, Newton per second; Freq, frequency; St⋅meter^–1^, strokes per meter; S, seconds; 1RM, one repetition maximum; W, watt. ^∗^*p* < 0.05 different from females. ^∗∗^*p* < 0.01 different from females.*

Independent of sex, strong correlations were found between 1RM pulldown and TT performance (*r*_xy_ = 0.83, *p* < 0.01) and maximal power in pulldown and performance (*r*_xy_ = 0.81, *p* < 0.01). The two variables CT (*r*_xy–z_ = 0.62, *p* < 0.01) and % of 1RM pull down during DP (*r*_xy–z_ = −0.56, *p* < 0.01) expressed the highest correlation with TT performance when corrected for sex. Within each sex, PF in DP (*r*_xy_ = −0.65, *p* < 0.05) among males, and CT (*r*_xy_ = 0.85, *p* < 0.01) among females expressed the highest correlations with TT performance. In Tables 3, 4, the potential relationships between test results and TT performance are presented both dependent and independent of sex, as well as within sexes.

**TABLE 3:** Correlations with time trial performance (*N* = 28).

	**Not corrected for sex**			**Corrected for sex**	
	***r*_xy_**	****SEE (%)****	*****p*****	*****r*_xy–z_****	*****p*****
VO_2max_ running					
L⋅min^–1^	–0.77	10.9	<0.01	–0.37	0.07
Ml⋅kg^–1^⋅min^–1^	–0.77	11.0	<0.01	–0.31	0.12
Ml⋅kg^–0.67^⋅min^–0.67^	–0.79	10.4	<0.01	–0.38	0.06
**VO_2peak_ DP**					
L⋅min^–1^	–0.78	10.7	<0.01	–0.42	0.05
Ml⋅kg^–1^⋅min^–1^	–0.77	11.0	<0.01	–0.38	0.03
Ml⋅kg^–0.67^⋅min^–0.67^	–0.80	10.4	<0.01	–0.44	0.02
**C_DP_**					
Ml⋅kg^–1^⋅meter^–1^	0.40	15.8	0.04	0.24	0.23
Ml⋅kg^–0.67^⋅meter^–1^	0.28	16.5	0.14	0.19	0.34
MAS (km h^–1^)	–0.80	10.3	<0.01	–0.48	0.01
**LT**					
%VO_2peak_	0.22	16.8	0.26	0.16	0.44
Km⋅h^–1^	–0.78	10.7	<0.01	–0.40	0.04
Km⋅h^–1^ calc. (MAS⋅%VO_2peak_)	–0.77	10.7	<0.01	–0.39	0.04
**Force_DP_**					
Peak (N)	–0.75	11.5	<0.01	–0.41	0.04
Average during CT (N)	–0.45	15.5	0.02	–0.19	0.33
RFD (N⋅s^–1^)	–0.42	15.4	0.03	–0.19	0.35
%of 1RM pull-down	–0.65	13.4	<0.01	–0.56	<0.01
**DP**					
Freq. at LT (St⋅meter^–1^)	0.55	14.3	0.01	–0.07	0.73
Freq. at LT (St⋅s^–1^)	–0.18	16.9	0.36	–0.48	0.01
CT (s)	0.69	12.5	<0.01	0.62	<0.01
**Maximal strength**					
1RM pull-down (kg)	–0.83	10.5	<0.01	–0.50	0.02
1RM leg-press (kg)	–0.53	15.3	0.01	–0.09	0.68
Power pull-down (W)	–0.81	10.7	<0.01	–0.49	0.02
Power leg-press (W)	–0.68	13.2	<0.01	–0.27	0.21

*Values are correlation coefficient (*r*), significant level (*p*), and standard error of the estimate (SEE). Kg, kilograms; VO_2max_, maximal oxygen uptake; L⋅min^–1^, liters per minute; Ml⋅kg^–1^⋅min^–1^, milliliters per kg BW per minute; Ml⋅kg^–0.67^⋅min^–0.67^, milliliters per kg BW raised to the power of 0.67 per minute; DP, double poling; VO_2peak_, peak oxygen uptake during DP; C_*DP*_, oxygen cost of DP at LT; Ml⋅kg^–1^⋅meter^–1^, milliliters per kg BW per meter; Ml⋅kg^–0.67^⋅meter^–1^, milliliters per kg BW raised to the power of 0.67 per meter; MAS, maximal aerobic speed calculated as peak oxygen uptake during DP divided on C_*DP*_; km⋅h^–1^, kilometers per hour; LT, lactate threshold; N, Newton; CT, contact time; RFD, rate of force development; N⋅s^–1^, Newton per second; Freq. % of 1RM pull-down, percentage of one repetition maximum in pull-down during one full DP cycle; Frequency. St⋅meter^–1^, strokes per meter; 1RM, one repetition maximum; W, watt.*

**TABLE 4:** Within sex correlations with time trial performance.

	**Males (*N* = 16)**		**Females (*N* = 12)**	
	***r*_xy_**	***p***	***r*_xy_**	***p***
**VO_2max_ running**				
L⋅min^–1^	–0.39	0.16	–0.49	0.11
Ml⋅kg^–1^⋅min^–1^	0.16	0.57	–0.70	0.01
Ml⋅kg^–0.67^⋅min^–0.67^	–0.10	0.71	–0.66	0.02
**VO_2peak_ DP**				
L⋅min^–1^	–0.57	0.02	–0.46	0.13
Ml⋅kg^–1^⋅min^–1^	–0.26	0.33	–0.51	0.09
Ml⋅kg^–0.67^⋅min^–0.67^	–0.43	0.09	–0.53	0.08
**C_DP_**				
Ml⋅kg^–1^⋅meter^–1^	0.53	0.03	0.07	0.82
Ml⋅kg^–0.67^⋅meter^–1^	0.40	0.12	0.08	0.81
MAS (km h^–1^)	–0.62	0.01	–0.56	0.06
**LT**				
%VO_2peak_	0.51	0.04	–0.27	0.40
Km⋅h^–1^	–0.18	0.51	–0.71	0.01
Km⋅h^–1^ calc. (MAS⋅%VO_2peak_)	–0.23	0.42	–0.74	0.01
**Force_DP_**				
Peak (N)	–0.65	0.01	–0.31	0.33
Average during CT (N)	–0.17	0.54	–0.26	0.41
RFD (N⋅s^–1^)	0.13	0.63	–0.38	0.22
% of 1RM pull-down	–0.44	0.10	–0.18	0.62
**DP**				
Freq. at LT (St⋅meter^–1^)	0.38	0.14	–0.23	0.47
Freq. at LT (St⋅s^–1^)	0.46	0.07	–0.75	0.01
CT (s)	–0.21	0.44	0.85	<0.01
**Maximal strength**				
1RM pull-down (kg)	–0.52	0.05	–0.52	0.12
1RM leg-press (kg)	–0.24	0.39	0.02	0.95
Power pull-down (W)	–0.47	0.08	–0.76	0.01
Power leg-press (W)	–0.27	0.34	–0.47	0.21

*Values are correlation coefficient (*r*) and significant level (*p*). Kg, kilograms; VO_2max_, maximal oxygen uptake; L⋅min^–1^, liters per minute; Ml⋅kg^–1^⋅min^–1^, milliliters per kg BW per minute; Ml⋅kg^–0.67^⋅min^–0.67^, milliliters per kg BW raised to the power of 0.67 per minute; DP, double poling; VO_2peak_, peak oxygen uptake during DP; C_*DP*_, oxygen cost of DP at LT; Ml⋅kg^–1^⋅meter^–1^, milliliters per kg BW per meter; Ml⋅kg^–0*.67*^⋅meter^–1^, milliliters per kg BW raised to the power of 0.67 per meter; MAS, maximal aerobic speed calculated as peak oxygen uptake during DP divided on C_*DP*_; km⋅h^–1^, kilometers per hour; LT, lactate threshold; N, Newton; CT, contact time; RFD, rate of force development; N⋅s^–1^, Newton per second; Freq. % of 1RM pull-down, percentage of one repetition maximum in pull-down during one full DP cycle; Frequency. St⋅meter^–1^, strokes per meter; 1RM, one repetition maximum; W, watt.*

The skiers with the highest 1RM pulldown also had the highest PF (*r*_xy_ = 0.78, *p* < 0.01) during DP. The same skiers also had the shortest CT (*r*_xy_ = 0.48, *p* < 0.05), and the lowest DP frequency measured as strokes pr. meter (*r*_xy_ = −0.71, *p* < 0.01). Relationships between maximal strength in pulldown and selected variables possibly related to maximal strength are presented in Table 5.

**TABLE 5:** Correlations with maximal strength in pull-down (*N* = 28).

	**Not corrected for sex**			**Corrected for sex**	
	***r*_xy_**	**SEE (%)**	***p***	***r*_xy–z_**	***p***
**C_DP_**					
Ml⋅kg^–1^⋅meter^–1^	−0.36	21.5	0.08	−0.18	0.40
Ml⋅kg^–0.67^⋅meter^–1^	−0.20	22.5	0.36	0.02	0.94
MAS (km h^–1^)	−0.74	15.2	<0.01	−0.21	0.32
**Force_DP_**					
Peak (N)	0.78	14.2	<0.01	0.50	0.01
Average during CT (N)	0.39	21.1	0.05	0.09	0.64
RFD (N⋅s^–1^)	0.31	21.8	0.13	0.02	0.92
% of 1RM pull-down	−0.54	19.3	0.01	−0.40	0.05
**DP**					
Freq. at LT (St⋅meter^–1^)	−0.71	16.1	<0.01	−0.20	0.35
Freq. at LT (St⋅s^–1^)	−0.09	22.9	0.66	0.08	0.72
CT (s)	−0.48	20.1	0.02	−0.29	0.17
**Maximal strength**					
Power pull-down (W)	0.92	8.9	<0.01	0.77	0.02

*Values are correlation coefficient (*r*), significant level (*p*), and standard error of the estimate (SEE). C_*DP*_, oxygen cost of DP at LT; Ml⋅kg^–1^⋅meter^–1^, milliliters per kg BW per meter; Ml⋅kg^–0.67^⋅meter^–1^, milliliters per kg BW raised to the power of 0.67 per meter; MAS, maximal aerobic speed calculated as peak oxygen uptake during DP divided on C_*DP*_; km⋅h^–1^, kilometers per hour; N, Newton; CT, contact time; RFD, rate of force development; N⋅s^–1^, Newton per second. % of 1RM pull-down, percentage of one repetition maximum in pull-down during one full DP cycle; Freq, frequency; St⋅meter^–1^, strokes per meter; 1RM, one repetition maximum; W, watt.*

## Discussion

The main findings in the present study are the correlations between roller skiing DPTT performance and maximal strength in pull-down, both independent and dependent of sex. Higher maximal upper body strength was related to higher PF in DP, lower DP frequency, and shorter CT.

The novelty of the present study was the finding of a strong correlation between maximal strength (1RM) in pulldown *per se* and roller skiing time trial performance in a cohort of competitive cross-country skiers from both sexes with the same relative training background and age, but with heterogeneity in performance.

### Correlations With TT Performance Independent of Sex

For the strength variables, strong correlations were found between 1RM pulldown and TT performance (*r*_xy_ = 0.83) and maximal power output in pulldown and performance (*r*_xy_ = 0.81). SEE was 10.5 and 10.7, respectively. The *r*^2^ values indicate that both variables predicts TT performance by 69%, and the SEE shows this to be outside a margin of approximately 10.5% of either 1RM or power output results. The 10.5% corresponds to 9 kg in pulldown. This implies that if one skier was at least 9 kg’s stronger than another in pulldown, he or she would perform better in TT. Regarding DP characteristics, PF (*r*_xy_ = −0.75), PF during DP as a percentage of 1RM (*r*_xy_ = −0.65) and CT during DP (*r*_xy_ = 0.69) correlated best with TT performance. The relationship between PF and TT is in accordance with previous studies demonstrating that faster skiers had higher PF, or that higher PF related to peak skiing speeds (Bilodeau et al., 1995; Holmberg et al., 2005; Stöggl and Holmberg, 2011, 2016; Stöggl et al., 2011). The two single physiological variables regarding aerobic endurance that correlated best with TT performance were VO_2__max_ in running and VO_2__peak_ DP expressed as ml⋅kg^–0.67^⋅min^–1^ (*r*_xy_ = 0.79 and *r*_xy_ = 0.80, respectively). The SEE value of 10.4%, implies that if one skier had at least 23 ml⋅kg^–0.67^⋅min^–1^ higher VO_2__peak_ DP than another, he or she would perform better in TT. There was also a strong correlation between velocity at LT and performance (*r*_xy_ = 0.78). All of these correlations were found in the heterogeneous cohort including both sexes.

### Correlations With TT Performance Corrected for Sex

When corrected for sex, the aerobic endurance variables decreased substantially in predicting TT performance. The two variables CT (*r*_xy–z_ = 0.62) and PF as a percentage of 1 RM pull down during DP (*r*_xy–z_ = −0.56) expressed the best correlation with TT performance when corrected for sex. When correcting for sex, the cohorts are more homogeneous since males and females results are clustered in to two groups. This was apparent when comparing coefficient of variance (CV) (Table 2). The CV for VO_2__max_ (running) and VO_2__peak_ DP when including both sexes, were both 18%. When separated into males and females, the CV values were cut in half. This phenomenon was not so obvious regarding strength and DP characteristics.

### Correlations With TT Performance Within Sexes

Both normal and partial correlations were performed in the present study. When partial correlations were still significant, this would strengthen the normal correlations by showing that it was not confounded by sex. However, the partial correlations only showed to what extent the normal correlations were confounded by sex, and so correlations with TT performance were also analyzed within sexes. These correlations should be handled with caution, due to the low number of skiers within each sex. The division into two separate groups also caused a greater degree of homogeneity in almost all variables. As a result of this and the low number in each group, correlations across sexes were weakened or disappeared. Those analyzes are merely included for informative reasons, but not addressed further in this discussion. Maximal strength in pulldown had approximately the same correlation with TT performance in both males and females. However, the relationships between the utilization of this maximal strength and TT performance seemed to differ, as CT correlated well in females but not males, and PF in DP correlated well in males but not females.

Both males and females had approximately the same correlation between MAS and TT performance. However, TT performance seemed to depend mostly on VO_2__peak_ but not C_DP_ in females. In males, TT performance seemed to depend mostly on C_DP_, but not VO_2__peak_.

### VO_2__max_, C_DP_, and MAS

The importance of a high VO_2__max_ in running for TT performance in the present study, is in accordance with several previous studies (Bergh, 1987; Ingjer, 1991; Mahood et al., 2001; Larsson et al., 2002; Alsobrook and Heil, 2009; Ainegren et al., 2013). This is also demonstrated by cross-country skiers’ high values of VO_2__max_; 80–90 ml⋅kg^–1^⋅min^–1^ and 70–80 ml⋅kg^–1^⋅min^–1^ for men and women world-class cross-country skiers, respectively (Sandbakk et al., 2014). The VO_2__max_ values in the present study were 53.8 ± 4.4 ml⋅kg^–1^⋅min^–1^ and 72.2 ± 5.1 ml⋅kg^–1^⋅min^–1^ for women and men, respectively. Regarding the skiers cost of skiing, the present study found that C_DP_ did not correlate well with TT performance (Table 2). The low impact of C_DP_ in the present study is further highlighted when including C_DP_ in the MAS equation (VO_2__peak_ DP/C_DP_). MAS did not correlate better with TT performance than VO_2__peak_ DP alone (*r*_xy_ = −0.80), independent of sex.

### Lactate Threshold

Maximal oxygen consumption at LT in% of VO_2__peak_ DP did not correlate with TT performance in the present study. However, velocity at LT correlated strongly (*r*_xy_ = −0.78) with TT performance. This is in accordance with Støren et al. (2014) on cyclists. In Støren et al. (2014) it was shown that velocity at LT, as measured in the present study, also could be calculated by the product of MAS and LT in% of VO_2__peak_, while LT in% of VO_2__peak_ alone did not explain LT velocity. The same results were echoed in the present study. When applying the same equation for velocity at LT (MAS LT%), this correlated nearly perfect (*r*_xy_ = 0.99) with the actually measured LT velocity. This implies that it is not LT *per se*, but rather VO_2__peak_ that predicts TT performance.

### Maximal Strength

Although C_DP_ correlated weakly with TT performance in the present study, variables previously shown to affect work economy in other sports (Saunders et al., 2004; Støren et al., 2008; Sunde et al., 2010), such as maximal strength and movement cycle characteristics correlated well with performance in the present study. This indicates that during DP the% of 1RM pulldown, CT, PF, and 1RM pulldown and power output in 1RM pulldown actually affected TT performance *per se* and not merely via C_DP_. That maximal strength *per se* is related to TT performance, is in contrast to previous studies in running and cycling (Støren et al., 2008, 2013). One possible explanation for this difference is that DP relies much more on upper body work than running and cycling. Blagrove et al. (2018) and Fletcher and MacIntosh (2017), discussed that an improvement in force-generating capacity would theoretically allow athletes to sustain a lower percentage of maximal strength during running. It is likely that this also applies to the cross-country skiers during DP in the present study. A higher 1RM pulldown would therefore imply a lower percentage of 1RM during DP, at a given work load. Thus the% of 1RM pull down correlated good to TT performance in the present study (*r*_xy_ = −0.65).

### Maximal Strength and DP Characteristics

The skiers with the highest 1RM pulldown also had the highest PF (*r*_xy_ = 0.78) during DP (Table 4). The same skiers also had the shortest CT (*r*_xy_ = 0.48), and the lowest DP frequency measured as strokes per meter (*r*_xy_ = −0.71). Also CT correlated with LT velocity (*r*_xy_ = −0.53), indicating that the fastest skiers had the shortest CT, although CT did not correlate with TT directly. Therefore, this shortened CT in faster skiers might be basically explained by the higher skiing speeds. A shorter CT and a lower frequency allows for a shorter contraction time and a longer transit time during each DP cycle. This could theoretically lead to better circulation and thus O_2_ and substrate deliverance as well as better clearance of lactic acid (Barrett-O’Keefe et al., 2012). On the other hand, the indication of the fastest skiers having the shortest CT, could partly explain the correlation between CT and 1RM pulldown.

### Upper Body vs. Lower Body Maximal Strength and Power Output

Even though DP may be considered a whole body exercise involving muscle mass from feet to neck, the leg press results in the present study seemed to have much less impact on TT performance and MAS than pulldown. This does not necessarily imply that lower body muscles do not have an impact on DP. Based on EMG activity in lower body muscles, Holmberg et al. (2005) showed that DP requires more than upper body work. Also power was measured for the strength variables in the present study. In pulldown, the impact of power more or less followed the impact of 1RM on TT performance and MAS. It is not surprising that the strongest also produced most power output, and this is in accordance with previous studies (Østerås et al., 2002).

Power output was calculated as the product of force and work distance divided by time. The power output results in leg press in the present study may seem low. This is due to the measurements of work distance being performed vertically when the lifting direction is diagonal in the leg press apparatus. In studies were the lifting direction is vertical like squat in e.g., Støren et al. (2008) and Sunde et al. (2010), power output results in runners and cyclist were shown to be approximately 100% higher than in the present study.

### Sex Differences

Since the male and female participants in the present study represented a higher and a lower TT performance level, a comparison of the results from the two sexes may be used to discuss the importance of factors predicting DP performance. The male and female skiers were at the same age, and being recruited from the same teams and high schools, their training background was relatively similar. TT performance was 23% better in males than in females. The sex difference in the present study was therefore in accordance with results from Sandbakk et al. (2014) in DP, finding a 20% sex difference in a ramp DP protocol to exhaustion. The 23% sex difference in the present study corresponded to a 30% higher MAS in males. As VO_2__peak_ can be expressed as ml.kg^–1^.min^–1^, and DP as ml.kg^–1^.m^–1^, the product of denominations equals m min^–1^, which may also be expressed as km.h^–1^. The gender difference in MAS, could therefore be explained by 18% difference in VO_2__p__eak_ DP, and a none significant 8% difference in C_DP_. These differences is somewhat lower than presented in Sandbakk and Holmberg (2017).

Males where 34% stronger in pulldown than females, which is a somewhat lower difference than the 50% reported in Sandbakk et al. (2014). An interesting question is to what extent this influence the sex differences in MAS. Although a strong correlation between MAS and pulldown exist, the correlation weakens when corrected for sex. This could imply that the correlation was merely a product of males both being stronger and having a higher MAS than females. However, we cannot rule out that the higher MAS in males were at least partly due to a higher strength in pull down. On the other hand, the relationship between 1RM pulldown and TT performance corrected for sex is better than the relationship between 1RM pulldown and MAS. This may indicate that maximal strength could be important independent of MAS.

### Practical Implications

The practical implications of the present study is to acknowledge maximal upper body strength as a possible performance determining factor in DP. We suggest including maximal strength training in the cross-country skiers training programs, but the effect of this needs further evaluation in future studies. We recommend few repetitions (2–5) in 3–5 series with maximal mobilization in the concentric phase, with relatively long (2–3 min) pauses in between. These principles have in previous studies been shown to improve work economy as well as maximal strength (Østerås et al., 2002; Støren et al., 2008; Sunde et al., 2010).

## Conclusion

In conclusion maximal upper body strength was shown to have a significant impact on DP roller skiing performance, both dependent and independent of sex, and both dependent and independent of C_DP_. Higher maximal upper body strength was related to higher DP peak forces, lower DP frequency and shorter CT.

## Data Availability

The datasets generated for this study are available on request to the corresponding author.

## Ethics Statement

This study was carried out in accordance with the recommendations of the Institutional Review Board (IRB) at the University of South-Eastern Norway with written informed consent from all subjects. All subjects gave written informed consent in accordance with the Declaration of Helsinki. The protocol was approved by the Institutional Review Board (IRB) at the University of South-Eastern Norway.

## Author Contributions

AS, J-MJ, ØS, JH, GP, and MB participated significantly in the planning and designing of the study. AS, J-MJ, ØS, and JH participated in the data analysis and writing of the manuscript. AS, ØS, J-MJ, and MG participated in the data collection and analysis. All authors read and approved the manuscript.

## Conflict of Interest Statement

JH was employed by the company Myworkout. The remaining authors declare that the research was conducted in the absence of any commercial or financial relationships that could be construed as a potential conflict of interest.

## Abbreviations

C_DP_: oxygen cost in double poling
CT: contact time
DP: double poling
HR: heart rate
HR_max_: maximal heart rate
LT: lactate threshold
MAS: maximal aerobic speed
PF: peak force
RER: respiratory exchange ratio
RER_peak_: peak respiratory exchange ratio
TT: double poling time trial
VO_2_: oxygen uptake
VO_2max_: maximal oxygen uptake in running
VO_2peak_ DP: peak oxygen uptake in double poling.
